# Extending tetrahedral network similarity to carbon: A type-I carbon clathrate stabilized by boron

**DOI:** 10.1126/sciadv.adv6867

**Published:** 2025-05-23

**Authors:** Timothy A. Strobel, Tiange Bi, Piotr A. Guńka, Mads F. Hansen, Julia-Maria Hübner, Samuel G. Dunning, Li Zhu, Stella Chariton, Vitali B. Prakapenka, Yue Meng

**Affiliations:** ^1^ Earth and Planets Laboratory, Carnegie Institution for Science, 5241 Broad Branch Road NW, Washington, DC 20015, USA.; ^2^ Faculty of Chemistry, Warsaw University of Technology, Noakowskiego 3, 00-664 Warszawa, Poland.; ^3^ Department of Physics, Rutgers University, Newark, NJ 07102, USA.; ^4^ Center for Advanced Radiation Sources, The University of Chicago, Chicago, IL 60439, USA.; ^5^ HPCAT, X-ray Science Division, Argonne National Laboratory, Lemont, IL 60439, USA.

## Abstract

Clathrates are guest/host framework compounds composed of polyhedral cages, yet despite their prevalence among tetrahedral network formers, clathrates with a carbon host lattice remain unrealized synthetic targets. Here, we report a type-I carbon-based framework—a ubiquitous clathrate structure type found throughout compounds containing tetrahedral building blocks. Following a boron-stabilization scheme based on first-principles predictions in the Ca–B–C system at high pressure, type-I Ca_8_B*_x_*C_46−__*x*_ (*x* ≈ 9) was synthesized in the archetypal *Pm*$\bar{3}$*n* lattice with stability derived from substitutionally disordered boron atoms on hexagonal ring framework positions. The synthesized clathrate, which is recoverable to ambient conditions, expands topological network similarity across tetrahedral systems and opens possibilities for a broad family of diamond-like, carbon-based compounds with tunable properties based on the wide potential for guest/host-atom substitutions and framework versatility.

## Teaser

A type-I carbon-based clathrate was stabilized with boron, extending structural similarity across tetrahedral network formers.

## INTRODUCTION

Clathrate compounds (*1*) with polyhedral cages that tile three-dimensional space populate tetrahedral systems including water (clathrate hydrates) (*2*), silica (zeolites/clathrasils) (*3*, *4*), group 14 elements (*5*–*9*), and III–V semiconductors (*10*). Various clathrate structures are distinguished by the arrangements and types of polyhedral cages that can trap guest atoms or small molecules. The most common clathrate structures found across all tetrahedral building blocks are type-I and type-II (also referred to as sI and sII). Type-I clathrate contains two small [5^12^] cages (pentagonal dodecahedra) and six larger [5^12^6^2^] cages (tetradecahedra) in a cubic unit cell with 46 framework atoms in space group *Pm*$\bar{3}$*n* (zeolite framework type MEP). Type-II clathrate contains 16 small [5^12^] cages and 8 larger [5^12^6^4^] cages (hexadecahedra) in a cubic unit cell with 136 framework atoms in space group *Fd*$\bar{3}$*m* (zeolite framework type MTN).

Given the similarities between carbon and the other group 14 elements, the formation of carbon clathrate structures was postulated after the discovery of type-I and type-II silicon clathrates in the 1960s (*5*, *11*). Nevertheless, these structures have never been made for carbon and represent challenging synthetic targets as low-energy metastable phases (*12*–*17*), though thermodynamic stability has been suggested under hydrostatic tension (*18*). Carbon clathrate structures thus represent a void in observed four-coordinate nets based on structural similarity of tetrahedral building blocks (*19*). If produced, carbon clathrates would serve as diamond-like tetrahedral frameworks with high hardness and strength (*20*–*22*). The tensile and shear strengths of type-I carbon clathrate are predicted to exceed those of diamond due to the spatial distribution of covalent bonds (*23*). Furthermore, the presence of guest atoms within the clathrate cages enables tunable electronic properties (*24*, *25*), including predictions of superconductivity (*26*–*29*), in addition to low-frequency rattling vibrations, which are important for low thermal conductivities in thermoelectric materials (*30*, *31*).

While all-carbon clathrate frameworks remain elusive to date, recent progress has been made by stabilizing carbon clathrate structures via boron substitution. The substitution of boron atoms was predicted to improve formation energies compared with all-carbon frameworks, and the first thermodynamically stable B–C clathrates were recently predicted and synthesized for SrB_3_C_3_ and LaB_3_C_3_ in the bipartite sodalite structure (type-VII clathrates) under high-pressure conditions (*32*–*34*). High pressure promotes the formation of *sp*^3^-bonded carbon, analogous to silicon and germanium at low pressure (e.g., the cubic diamond structure). Type-VII carbon–boron clathrates are low-compressibility *sp*^3^ frameworks with tunable electronic structures ranging from semiconductors to metals and superconductors depending on the composition of guest atoms (*33*–*37*). Recent predictions for structures with binary mixed guest atoms suggest potential for high-*T*_c_ superconductivity with transition temperatures approaching 100 K (*38*–*43*).

Boron-substituted carbon clathrates represent a promising class of materials with a large range of physical properties, and the successful synthesis of type-VII structures hints at the possibility of accessing the more common clathrate structure types found among other tetrahedral systems. Here, following first-principles calculations of thermodynamic stability at high pressure (*44*), we report the successful synthesis of a type-I carbon clathrate phase that is stabilized by boron and traps calcium ions in the large [5^12^6^2^] and small [5^12^] cages.

## RESULTS

**Fig. 1.**
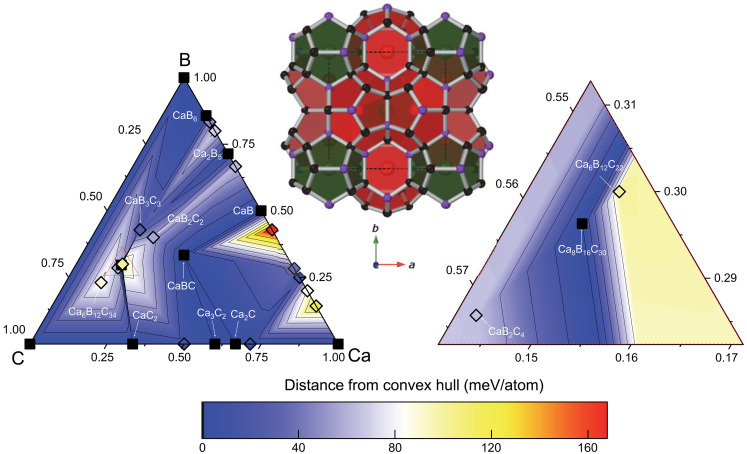
Calculated ternary Ca–B–C phase diagram at 50 GPa. Thermodynamically stable phases (located on the convex hull) are represented by black squares whereas metastable phases are represented by diamonds colored by their energy distance from the convex hull. The region within the red dashed triangle (left) is magnified (right) to show thermodynamically stable type-I Ca_8_B_16_C_30_ (space group *R*3). The rhombohedral Ca_8_B_16_C_30_ structure is shown with carbon and boron atoms as black and purple spheres, respectively. Large and small cages containing Ca atoms are colored red and green for visual distinguishability.

Motivated by predictions of thermodynamically stable Ca_8_B_16_C_30_ (Fig. 1) (*44*), the experimental synthesis of this phase was targeted at ~50 GPa using laser-heated diamond anvil cells (DACs) combined with in situ synchrotron x-ray diffraction (XRD) for structural characterization. After heating compressed precursor samples at temperatures above ~2500 K, new diffraction peaks appeared that were readily indexed to the cubic type-I clathrate lattice (*a* = 7.046 Å at ~50 GPa), in addition to cubic type-VII CaB_3_C_3_ clathrate (*a* = 4.529 Å, isostructural with SrB_3_C_3_) (*33*). Prolonged heating above 3000 K produced grains that were suitable for single-crystal diffraction methods.

Single-crystal structure solution revealed Ca atoms located on the 2*a* and 6*d* positions, and unambiguously revealed the type-I *Pm*$\bar{3}$*n* clathrate with framework atoms located on the 6*c*, 16*i*, and 24*k* positions. The initial all-carbon framework solution provides excellent structural refinement indicators (*R*_1_ = 0.035); however, this model (i.e., Ca_8_C_46_) is energetically implausible with a calculated convex hull distance > 700 meV/atom at 50 GPa. Furthermore, Ca_8_C_46_ is dynamically unstable with imaginary phonon frequencies throughout the first Brillouin zone (see fig. S1 and the Supplementary Materials). Thus, boron atoms must substitute within the type-I framework for stability, and the high quality of the all-carbon structural model reflects the difficulty in distinguishing carbon from boron (coloring) using XRD given the one-electron differential between the elements (*45*, *46*). After systematic refinement of potential boron distribution schemes in the cubic unit cell, a model with partial boron occupation of the 24*k* site provided the best structural refinement indicators (*R*_1_ = 0.032) with the composition Ca_8_B_8.9(14)_C_37.1 (14)_ (1σ standard uncertainty), or Ca_8_B_9 ± 3_C_37 ± 3_ with 95% probability (2σ level confidence interval). Boron occupation of the 16*i* site is disfavored, leading to unphysical occupancy factors in all cases, including the simultaneous refinement of all three framework site occupancies. A small fraction of boron on the 6*c* site, in addition to 24*k*, is conceivable but shows statistically insignificant occupancy (*R*_1_ = 0.033), and with partial occupation of both sites, the estimated uncertainty in the refined B composition, *x*, ranges between 7 ≤ *x* ≤ 15 with 95% probability. Structure refinement models are described in the Supplementary Materials and parameters are shown in tables S1 and S2. Supplementary crystallographic data are available for this paper (see the data and materials availability statement).

The experimentally observed structure is in good agreement with the thermodynamically stable phase from computational predictions. A key difference is the requirement of ordered boron decorations in the static calculations, which necessitate a lowering of symmetry from cubic (*Pm*$\bar{3}$*n*) to rhombohedral (*R*3) for the unit cell to accommodate the boron atoms on distinct sites. Partial site occupancies are used to treat the statistical distribution of boron substitutions empirically. Nevertheless, the calculated rhombohedral distortion is miniscule with *a* = 7.03 Å and α = 89.99° for the optimized cell at 50 GPa (cf., *a* = 7.05 Å and α, β, γ = 90° from experiment), and the predicted structure can be approximated as cubic, but with ordered Wyckoff positions.

**Fig. 2.**
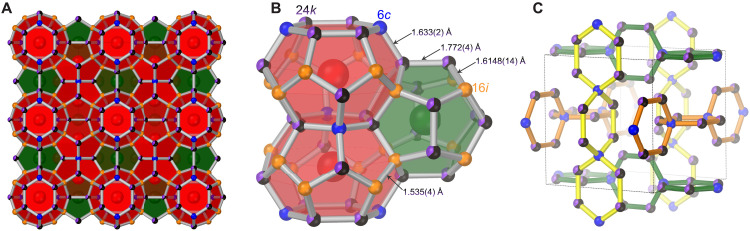
Structure of type-I Ca_8_B*_x_*C_46-__*x*_. (**A**) Packing of small (green) and large (red) polyhedral cages. (**B**) Detailed cage connectivity. Carbon atoms located on the 6*c* and 16*i* sites are represented by blue and orange spheres, respectively. Boron atoms that are substitutionally disordered with carbon on the 24*k* site are represented by purple/black pie-chart spheres proportional to partial occupancies. Bond distances between C/B and C/C sites are indicated. (**C**) Hexagonal ring chains (distinguished by bond colors) contain B atoms on the 24*k* site.

The experimentally determined type-I Ca–B–C clathrate structure is shown in Fig. 2. Similar to the predicted stable *R*3 Ca_8_B_16_C_30_ structure, boron atoms in the experimental *Pm*$\bar{3}$*n* lattice are primarily located on the 24*k* Wyckoff position. [Note that the 3*a* and 9*b* sites of *R*3 are related back to the three framework positions found in the parent structure, as described in (*44*).] Boron occupation of the 24*k* site in the experimental structure is clearly reflected in average bond distances. Average distances containing B–C contacts are longer [24*k*–24*k* = 1.772(4) Å, 24*k*–6*c* = 1.633(2) Å, 24*k*–16*i* =1.6148(14) Å] than distances that are exclusively C–C [16*i*–16*i* =1.535(4) Å]. The 24*k* and 6*c* Wyckoff positions are associated with the hexagonal rings of the large [5^12^6^2^] cages. The 6*c* site is exclusive to the large cages, while sites of the hexagons associated with the 24*k* sites are common between small cages. Chains of perpendicular hexagonal rings (rotated 90° about the 6*c* position) run parallel along all opposing faces of the unit cell. Boron occupation of the hexagonal rings allows for the minimization of bond angle strain for the ~120° hexagonal angle compared with the ideal 109.5° angle for tetrahedral carbon, in excellent agreement with model calculations (*44*). It is notable that Al doping within type-I silicon clathrates follows a similar substitution scheme, whereas B doping in Si clathrates occurs predominantly on the 16*i* positions due to size mismatch (*47*–*50*).

## DISCUSSION

On the basis of initial computational design requirements for charge balance (i.e., 2B per Ca), the theoretical *R*3 Ca_8_B_16_C_30_ structure is an insulating phase with a calculated density functional theory bandgap of ~2.5 eV (*44*). While single-crystal diffraction results unambiguously confirm the cubic type-I clathrate structure with boron occupation on 24*k*, refinements of substitutionally disordered boron in the *Pm*$\bar{3}$*n* lattice apparently violate this charge balance, although uncertainty is associated with the XRD-derived boron composition. That is, the highest reliability structural refinements suggest only ~6 to 12 boron atoms per unit cell, which cannot entirely compensate charge from fully occupied calcium ions. Future electrical transport measurements on phase-pure samples (the coexisting CaB_3_C_3_ phase is metallic) will elucidate the electronic structure of the type-I clathrate and potential relevance to high-*T*_c_ superconductivity as in related materials (*28*, *36*, *41*). Many clathrate compounds of heavier tetrel elements are known to deviate from precise electron counts (*33*, *51*–*53*). The possibility for substitutions of a variety of guest ions within the large and small cages (e.g., mono/trivalent), in combination with different framework coloring schemes, provides wide potential to systematically tune the physical properties of these compounds.

**Fig. 3.**
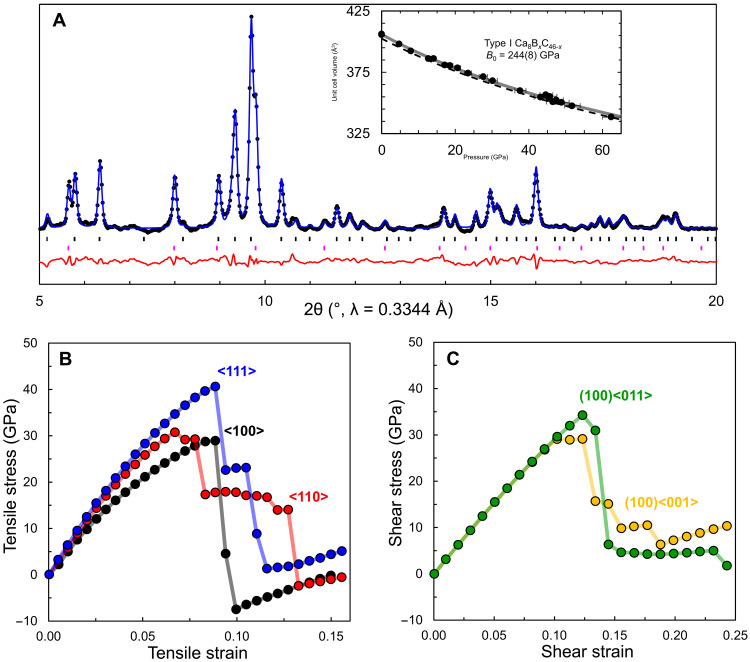
Recoverability and mechanical properties. (**A**) Powder XRD data (points), Rietveld refinement (blue line), and residual (red line) of type-I Ca_8_B*_x_*C_46-*x*_ clathrate recovered to ambient pressure. The type-I clathrate (upper tick marks, 73 wt %) coexists with type-VII CaB_3_C_3_ clathrate (lower tick marks, 23 wt %). The inset shows the experimental type-I clathrate unit cell volume as a function of pressure (points) and the refined *PV* equation of state (solid line) compared with the theoretical equation of state for ordered type-I Ca_8_B_16_C_30_ (dashed line). Calculated tensile strength (**B**) and shear strength (**C**) for Ca_8_B_16_C_30_ at ambient pressure.

Powder XRD measurements obtained during decompression indicate that the clathrate is recoverable to ambient conditions and persists in air for the maximum duration tested (several hours during synchrotron time). Rietveld refinement of powder data obtained at ambient pressure reveals only minor perturbations from the structural model obtained at ~50 GPa, showing an expanded lattice parameter of *a* = 7.4040(2) Å at 1 atm (Fig. 3A). Pressure-dependent refinements of the type-I unit cell volume and equation of state analysis revealed a zero-pressure bulk modulus *B*_0_ = 244(8) GPa, in good agreement with the calculated value for *R*3 Ca_8_B_16_C_30_ (*B*_0_ = 256 GPa). This agreement suggests that calculated structural properties of the *R*3 model are largely representative of the experimentally observed cubic cell. The type-I compressibility is similar to type-VII SrB_3_C_3_ and LaB_3_C_3_ clathrates, and ~14% larger than that of CaB_3_C_3_ [experimental *B*_0_ = 214(10) GPa; calculated *B*_0_ = 224 GPa] (*33*, *34*). On the basis of first-principles calculations, the tensile strength of the type-I clathrate is estimated to be ~30 GPa for the weakest direction along <100>, with a similar magnitude of the shear strength, which is weakest for {100} sheared along <001> (Fig. 3, B and C). These values are suggestive of a Vickers hardness (*54*) near ~30 GPa—in agreement with semi-empirical model estimates (*55*, *56*) between 31 and 39 GPa—placing the clathrate on the cusp of known superhard materials with advanced mechanical properties similar to those of boron carbide (*57*). The introduction of Ca and B into the framework substantially alters the mechanical properties of the clathrate as compared with hypothetical all-carbon C_46_. The bulk modulus of the pure allotrope is calculated to be ~400 GPa and the tensile and shear strengths are both estimated to exceed 100 GPa (*21*, *23*). Nevertheless, the incorporation of boron enables the thermodynamic synthesis of the type-I framework, and the introduction of a variety of guest atoms holds potential to access a wide range of electronic properties while maintaining a robust covalent lattice.

Following theoretical predictions of a thermodynamically stable type-I clathrate phase, high-pressure synthesis has revealed a carbon-based type-I clathrate, closing the gap for observed structural network topologies among tetrahedral building blocks. Boron atoms serve to stabilize the clathrate framework via partial substitution with carbon on hexagonal ring positions, and account for approximately 20% of the framework composition. The boron-stabilized type-I structure remains highly incompressible with large tensile and shear strengths, comparable to advanced technical ceramics. Moreover, a wide range of potential host/guest substitutions provides the opportunity to readily modify the electronic structure and optimize synthetic conditions to create a broad family of tunable diamond-like framework materials.

## MATERIALS AND METHODS

### Experimental synthesis

Precursor powders were prepared by ball milling CaB_6_ (Sigma-Aldrich, 99.5%), CaC_2_ (>98%, prepared following a literature method) (*58*), and glassy carbon (Sigma-Aldrich, 99.95%) under argon at 600 rpm for 99 1-min cycles, targeting a bulk composition of Ca_8_B_16_C_30_. The milled powder was pressed into ~50 μm × 50 μm × 10 μm pellets using 1-mm flat diamond anvils, and the pellets were loaded into DACs equipped with 300-μm culets and ~40-μm–thick Re gaskets with ~150-μm–diameter sample chambers. All sample pellets were loaded within an inert Ar glovebox with O_2_/H_2_O < 1 parts per million. The sample chambers were subsequently loaded and clamped with Ne at ~1 kbar, which served as thermal insulation, the pressure medium, and XRD pressure calibrant (*59*).

The DAC samples were compressed to ~50 GPa and heated with a double-sided infrared laser system with in situ synchrotron XRD at the Advanced Photon Source, sector 13 [GeoSoilEnviroCARS (GSECARS)] and sector 16 [High-Pressure Collaborative Access Team (HPCAT)] (*60*, *61*). Diffraction data were collected using a Pilatus3 CdTe 1M hybrid photon counting detector, which was calibrated using LaB_6_/CeO_2_ powder standards and an enstatite single-crystal standard. After high-pressure, high-temperature synthesis, powder XRD patterns were obtained from samples during decompression to establish the *PV* equation of state (third-order Birch–Murnaghan) using EoSFit (*62*). Type-I samples were measured at ambient conditions in air and did not decompose over a period of hours. Powder data were analyzed using Dioptas and GSAS/EXPGUI (*63*, *64*).

### Single-crystal XRD

A multigrain sample was recrystallized from powder at 48(2) GPa using the laser heating system at GSECARS. Prolonged heating at >3000 K led to the formation of grains large enough to obtain diffraction patterns amenable to multigrain analysis with a synchrotron beam size < 5 μm × 5 μm. Frames were recorded between ω = −32° and +32° in 0.5° steps with an exposure time of 5 or 3 s per frame. Reflections were harvested using CrysAlis^PRO^ (*65*), assigned to individual domains with DAFi (*66*), and subsequently indexed and integrated using the CrysAlis^PRO^ software suite. Crystal structures were solved with SHELXT 2018/2 and refined using SHELXL 2019/3, invoked from within the Olex2 suite (*67*–*69*). Reflections from three individual grains were merged using SORTAV for structure refinement of type-I clathrate (*70*, *71*). SORTAV was invoked from WinGX (*69*). In the case of type-VII clathrate, diffraction data from one grain were used.

### Computational methods

Calculations were performed in the framework of density functional theory within the Perdew–Burke–Ernzerhof (*72*) parameterization of the generalized gradient approximation (*73*) as implemented in the VASP (Vienna Ab Initio simulation package) code (*74*). The projector-augmented wave (PAW) method (*75*) was adopted with the PAW potentials taken from the VASP library where 3*p*^6^4*s*^2^, 2*s*^2^2*p*^1^ and 2*s*^2^2*p*^2^ are treated as valence electrons for Ca, B, and C atoms, respectively. A plane-wave kinetic energy cutoff of 500 eV was used for convergence of total energies. The dynamical stability of structures was established via phonon dispersion calculations using the finite displacement approach, as implemented in the Phonopy code (*76*). Stress–strain relations were calculated by estimating the stress response to structural deformation along specific loading paths using a quasistatic relaxation method (*77*). The stress response under tensile and shear strains was used to establish the ideal strengths, i.e., the lowest stress to plastically deform a perfect crystal.

## Supplementary Materials

### This PDF file includes:

Description of structural models Tables S1 to S3 Figs. S1 to S3
