# Supplementary material for: Extending tetrahedral network similarity to carbon: A type-I carbon clathrate stabilized by boron
Source: Sci Adv. 2025 May 23;11(21):eadv6867. doi: 10.1126/sciadv.adv6867 (PMC12101512; doi:10.1126/sciadv.adv6867)
Supplement: Supplementary file 1 — Description of structural models Tables S1 to S3 Figs. S1 to S3 [file sciadv.adv6867_sm.pdf]

Science Advances
AAAS

Supplementary Materials for

# Extending tetrahedral network similarity to carbon: A type-I carbon clathrate stabilized by boron

Timothy A. Strobel *et al.*

Corresponding author: Timothy A. Strobel, tstrobel@carnegiescience.edu

*Sci. Adv.* **11**, eadv6867 (2025)
DOI: 10.1126/sciadv.adv6867

**This PDF file includes:**

Description of structural models
Tables S1 to S3
Figs. S1 to S3

*Description of crystal structure models*

**Table S1** contains crystal data and structure refinement parameters for type-I $Ca_8B_xC_{46-x}$ and type-VII $CaB_3C_3$. The initial crystal structure solution revealed fully occupied Ca atoms located on the 2*a* and 6*d* positions as well as clathrate framework atoms on the 6*c*, 16*i,* and 24*k* positions. Initially, only models with ordered framework atoms were refined (see models **1** and **2a**-**2c** in **Table S2**). It was found that the pure-carbon framework model (**1**) gives the best refinement indicators among the ordered models. However, theoretical computations have shown that $Ca_8C_{46}$ is energetically and dynamically unstable. Hence, refinement of disordered arrangements of C/B atoms was attempted. It was found that the introduction of C/B disorder on the 16*i* leads to unphysical negative boron occupancy in all cases, including two-and three-site disorder, while disorder on 6*c* does not significantly change the boron occupancy from zero (models **3a** and **3c** in **Table S2**). Only the introduction of disorder on the 24*k* position (model **3b** in **Table S2**) leads to significant improvement of refinement indicators and to a boron occupancy significantly different from zero (see **Table S2**). Introduction of B/C disorder on both 24*k* and 6*c* positions (model **3d**) does not lead to improvement of refinement indicators compared with model **3b** and the occupancy of B on the 6*c* position does not differ significantly from zero. Therefore, model **3b** is preferred to model **3d**. Finally, anisotropic displacement parameters for clathrate framework atoms have been refined (model **4** in **Table S2**).

**Table S1.** Single-crystal refinement parameters for type-I and type-VII Ca–B–C clathrates at 48(2) and 52(2) GPa, respectively.

| Empirical formula | $Ca_8B_{8.9±1.4}C_{37.1±1.4}$ | $CaB_3C_3$ |
|---|---|---|
| CSD number | 2338100 | 2338099 |
| Formula weight | 862.55 | 108.54 |
| $T$/K | 293(2) | 293(2) |
| Crystal system | cubic | cubic |
| Space group | $Pm\bar{3}n$ | $Pm\bar{3}n$ |
| $a$ /Å | 7.0464(12) | 4.529(2) |
| $V$ /Å$^3$ | 349.87(18) | 92.89(14) |
| $Z$ | 1 | 2 |
| $\rho_{calc}$ /g·cm$^{-3}$ | 4.094 | 3.881 |
| $\mu$ /mm$^{-1}$ | 0.299 | 0.370 |
| $F$(000) | 427.1 | 106 |
| Crystal size*/mm3* | 0.005 × 0.005 × 0.005 | 0.005 × 0.005 × 0.005 |
| Radiation | Synchrotron (λ = 0.2952 Å) | (λ = 0.3344 Å) |
| 2Θ range for data collection/° | 3.40 to 30.2 | 5.99 to 30.0 |
| Index ranges | $-11 \leq h \leq 10$, $-12 \leq k \leq 11$, $-11 \leq l \leq 11$ | $-2 \leq h \leq 3$, $-6 \leq k \leq 6$, $-6 \leq l \leq 6$ |
| Reflections collected | 2324 | 107 |
| Independent reflections | 195 [$R_{int}$ = 0.059, $R_{sigma}$ = 0.030] | 28 [$R_{int}$ = 0.1815, $R_{sigma}$ = 0.0512] |
| Data/restraints/parameters | 195/0/16 | 28/0/3 |
| Goodness−of−fit on $F^2$ | 1.15 | 1.26 |
| Final $R$ indexes [$I$>=2$\sigma$($I$)] | $R_1$ = 0.032, $wR_2$ = 0.066 | $R_1$ = 0.085, $wR_2$ = 0.221 |
| Final $R$ indexes [all data] | $R_1$ = 0.046, $wR_2$ = 0.072 | $R_1$ = 0.096, $wR_2$ = 0.242 |
| Largest diff. peak/hole / eÅ$^{-3}$ | +0.62/−0.53 | +1.8/−1.14 |

**Table S2.** Comparison of different structural models for type-I Ca–B–C clathrate.

| data/model | **1** | **2a** | **2b** | **2c** | **3a** | **3b** | **3c** | **3d** | **4** |
|---|---|---|---|---|---|---|---|---|---|
| Atom positions | Ca (2*a*)<br>Ca (6*d*)<br>C (16*i*)<br>C (24*k*)<br>C (6c) | Ca (2*a*)<br>Ca (6*d*)<br>B (16*i*)<br>C (24*k*)<br>C (6c) | Ca (2*a*)<br>Ca (6*d*)<br>C (16*i*)<br>B (24*k*)<br>C (6c) | Ca (2*a*)<br>Ca (6*d*)<br>C (16*i*)<br>C (24*k*)<br>B (6c) | Ca (2*a*)<br>Ca (6*d*)<br>B/C (16*i*)<br>C (24*k*)<br>C (6c) | Ca (2*a*)<br>Ca (6*d*)<br>C (16*i*)<br>B/C (24*k*)<br>C (6c) | Ca (2*a*)<br>Ca (6*d*)<br>C (16*i*)<br>C (24*k*)<br>B/C (6c) | Ca (2*a*)<br>Ca (6*d*)<br>C (16*i*)<br>B/C (24*k*)<br>B/C (6c) | Ca (2*a*)<br>Ca (6*d*)<br>C (16*i*)<br>B/C (24*k*)<br>C (6c) |
| Reflections collected | 2324 [$R_{int}$ = 0.059, $R_{sigma}$ = 0.030] | | | | | | | | |
| Independent reflections | 195 | | | | | | | | |
| Data/restraints/parameters | 195/0/10 | | | | 195/0/11 | | | 195/0/12 | 195/0/16 |
| Chemical formula | $Ca_8C_{46}$ | $Ca_8B_{16}C_{30}$ | $Ca_8B_{24}C_{22}$ | $Ca_8B_6C_{40}$ | $Ca_4B_{-0.2}C_{23}$ | $Ca_8B_{9.12}C_{36.88}$ | $Ca_8B_{0.78}C_{45.22}$ | $Ca_8B_{10.92}C_{35.08}$ | $Ca_8B_{8.88}C_{37.12}$ |
| $10^3 \cdot U_{11}$ (Ca (2*a*)) /Å$^2$ | 3.7(3) | 3.4(5) | 3.9(3) | 3.8(3) | 3.8(3) | 3.9(3) | 3.7(3) | 3.9(3) | 3.8(3) |
| $10^3 \cdot U_{11}$ (Ca (6*d*)) /Å$^2$ | 4.0(4) | 3.6(6) | 4.0(4) | 4.0(4) | 3.9(4) | 4.1(4) | 4.0(4) | 4.1(3) | 4.2(3) |
| $10^3 \cdot U_{33}$ (Ca (6*d*)) /Å$^2$ | 6.0(3) | 5.9(5) | 6.2(3) | 6.0(3) | 6.1(3) | 6.1(2) | 6.0(3) | 6.1(2) | 6.1(2) |
| $10^3 \cdot U_{iso}$ (C/B (16*i*)) /Å$^2$ | 5.7(4) | 1.9(7) | 6.2(5) | 5.8(5) | 6.9(5) | 5.7(4) | 5.7(4) | 5.8(4) | 5.7(4)$^\dagger$ |
| $10^3 \cdot U_{iso}$ (C/B (24*k*)) /Å$^2$ | 6.8(4) | 6.6(6) | 3.2(4) | 6.8(4) | 6.7(4) | 5.5(4) | 6.8(4) | 5.4(4) | 5.5(4)$^\dagger$ |
| $10^3 \cdot U_{iso}$ (C/B (6*c*)) /Å$^2$ | 7.2(7) | 7.4(12) | 7.2(8) | 3.7(8) | 7.2(7) | 7.2(6) | 6.7(8) | 6.4(7) | 7.6(6)$^\dagger$ |
| B molar fraction* | - | - | - | - | <0$^\ddagger$ | 0.38(6) | 0.13(12) | 0.40(6) – 24*k*<br>0.22(11) – 6*c* | 0.37(6) |
| Goodness-of-fit on $F^2$ | 1.12 | 1.26 | 1.19 | 1.19 | 0.843 | 1.14 | 1.14 | 1.13 | 1.15 |
| Final *R* indexes [$I \geq 2\sigma(I)$] | $R_1$ = 0.035, $wR_2$ = 0.075 | $R_1$ = 0.045, $wR_2$ = 0.136 | $R_1$ = 0.037, $wR_2$ = 0.093 | $R_1$ = 0.038, $wR_2$ = 0.089 | $R_1$ = 0.036, $wR_2$ = 0.086 | $R_1$ = 0.033, $wR_2$ = 0.069 | $R_1$ = 0.035, $wR_2$ = 0.074 | $R_1$ = 0.033, $wR_2$ = 0.069 | $R_1$ = 0.032, $wR_2$ = 0.066 |
| Final *R* indexes [all data] | $R_1$ = 0.049, $wR_2$ = 0.082 | $R_1$ = 0.060, $wR_2$ = 0.147 | $R_1$ = 0.052, $wR_2$ = 0.103 | $R_1$ = 0.053, $wR_2$ = 0.096 | $R_1$ = 0.049, $wR_2$ = 0.098 | $R_1$ = 0.047, $wR_2$ = 0.075 | $R_1$ = 0.049, $wR_2$ = 0.080 | $R_1$ = 0.047, $wR_2$ = 0.076 | $R_1$ = 0.046, $wR_2$ = 0.072 |
| $\rho_{max}/\rho_{min}$ / eÅ$^{-3}$ | +0.62/−0.65 | +1.2/−0.66 | +0.79/−0.61 | +1.2/−0.77 | +0.61/−0.68 | +0.62/−0.53 | +0.61/−0.66 | +0.61/−0.52 | +0.62/−0.53 |

* B molar fraction on the disordered site only; † $U_{eq}$ since C and B atoms were refined anisotropically. ‡All refinements of B on 16*i* produce unphysical parameters.

**Table S3.** Rietveld refinement parameters for type-I Ca–B–C clathrate at ambient pressure.

| Atom | Site | *x* | *y* | *z* | *Fractn* | *Uiso*×100 |
|---|---|---|---|---|---|---|
| Ca1 | 2*a* | 0 | 0 | 0 | 1 | 1.71(6) |
| Ca2 | 6*d* | 0.25 | 0.5 | 0 | 1 | 1.71(6) |
| C1 | 6*c* | 0.5 | 0.25 | 0 | 1 | 1.1(1) |
| C2 | 16*i* | 0.1871(5) | 0.1871(5) | 0.1871(5) | 1 | 1.1(1) |
| C3 | 24*k* | 0.3058(9) | 0.1274(6) | 0 | 0.62(3)* | 1.1(1) |
| B3 | 24*k* | 0.3058(9) | 0.1274(6) | 0 | 0.38(3)* | 1.1(1) |
| Space group | $Pm\bar{3}n$ | | | | | |
| *a* /Å | 7.4040(2) | | | | | |
| Radiation | Synchrotron (λ = 0.3344 Å) | | | | | |
| $R_{wp}$–Bknd | 0.022 | | | | | |

**Initialized composition from high-pressure SXRD data*

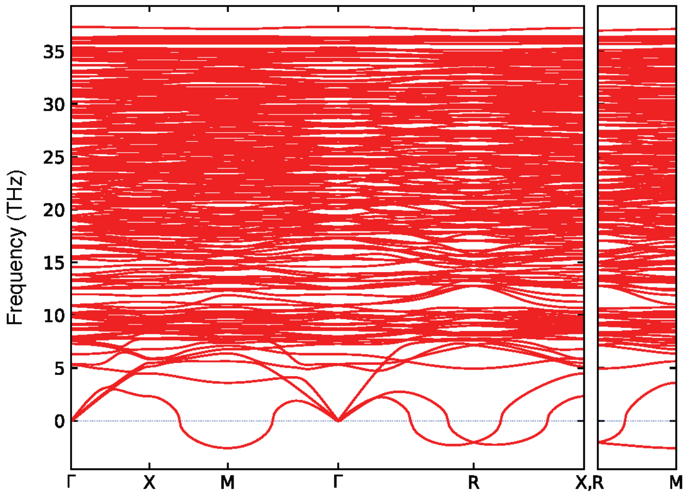


**Fig. S1.** Phonon dispersion relations for type-I $Ca_8C_{46}$ at 50 GPa showing that the structure is dynamically unstable without boron. The calculated convex hull distance is >700 meV/atom at 50 GPa.

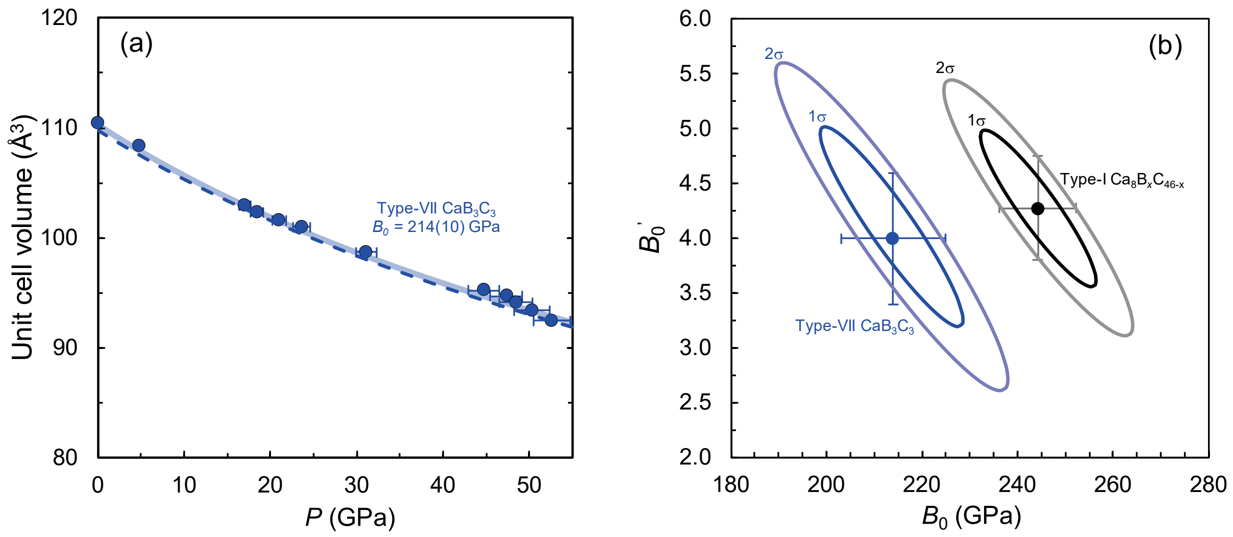


**Fig. S2.** Experimental *PV* data were modelled using a third-order Birch–Murnaghan equation of state (EOS) to obtain the zero-pressure volume, $V_0$, bulk modulus, $B_0$, and its pressure derivative, $B_0$'. Refined experimental parameters are $V_0$ = 405.9(3), $B_0$ = 244(8) and $B_0$' = 4.2(4) for type-I $Ca_8B_xC_{46-x}$ and $V_0$ = 110.5(1), $B_0$ = 214(10) and $B_0$' = 4.1(6) for type-VII $CaB_3C_3$, which compare favorably with DFT (PBE) calculations that yield $B_0$ = 256 and $B_0$' = 3.8 for type-I $Ca_8B_xC_{46-x}$ and $B_0$ = 224 and $B_0$' = 3.7 for type-VII $CaB_3C_3$. (a) Experimental unit cell volume as a function of pressure for type-I clathrate (points) and refined EOS (solid line) compared with theoretical equation of state for ordered type-I $Ca_8B_{16}C_{30}$ (dashed line). (b) Experimental uncertainty in $B_0$ and $B_0$' as confidence ellipses drawn at one and two standard deviations for type-I $Ca_8B_xC_{46-x}$ (black) and type-VII $CaB_3C_3$ (blue).

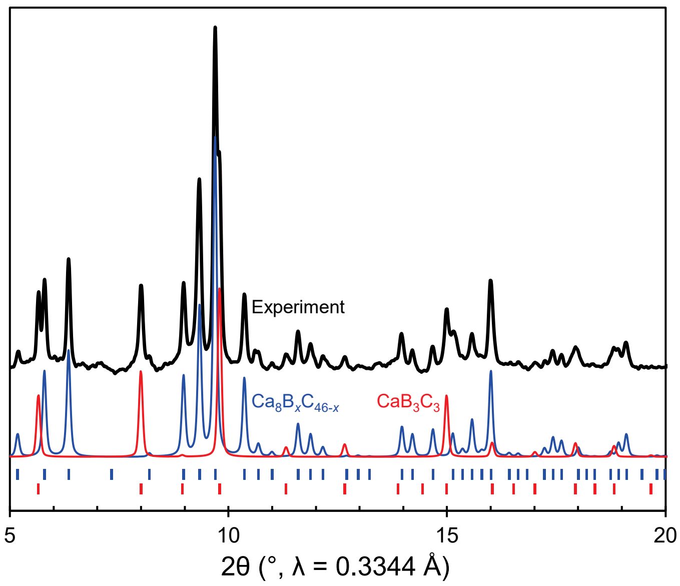


**Fig. S3.** Experimental powder XRD pattern (black) collected at ambient pressure (recovered from ~50 GPa) compared with calculated profiles for $Ca_8B_xC_{46-x}$ (red) and $CaB_3C_3$ (blue).
